# Supplementary material for: Realization of monolayer ZrTe5 topological insulators with wide band gaps
Source: Nat Commun. 2024 Jun 5;15:4784. doi: 10.1038/s41467-024-49197-x (PMC11153644; doi:10.1038/s41467-024-49197-x)
Supplement: Supplementary file 1 — Supplementary Information [file 41467_2024_49197_MOESM1_ESM.pdf]

Supplementary Information

"Realization of monolayer $ZrTe_5$ topological insulators with wide band gaps"

Yong-Jie Xu[1,†], Guohua Cao[2,†], Qi-Yuan Li[1], Cheng-Long Xue[1], Wei-Min Zhao[1], Qi-Wei Wang[1], Li-Guo Dou[1], Xuan Du[1], Yu-Xin Meng[1], Yuan-Kun Wang[1], Yu-Hang Gao[1], Zhen-Yu Jia[1], Wei Li[3], Lianlian Ji[3], Fang-Sen Li[3], Zhenyu Zhang[2,4], Ping Cui[2,4,*], Dingyu Xing[1,5], Shao-Chun Li[1,4,5,6,*]

[1]*National Laboratory of Solid State Microstructures, School of Physics, Nanjing University, Nanjing 210093, China*

[2]*International Center for Quantum Design of Functional Materials (ICQD), University of Science and Technology of China, Hefei 230026, China*

[3]*Vacuum Interconnected Nanotech Workstation, Suzhou Institute of Nano-Tech and Nano-Bionics, Chinese Academy of Sciences, Suzhou 215123, China*

[4]*Hefei National Laboratory, Hefei 230088, China*

[5]*Collaborative Innovation Center of Advanced Microstructures, Nanjing University, Nanjing 210093, China*

[6]*Jiangsu Provincial Key Laboratory for Nanotechnology, Nanjing University, Nanjing 210093, China*

[†] These authors contributed equally to this work.

* email: cuipg@ustc.edu.cn; scli@nju.edu.cn

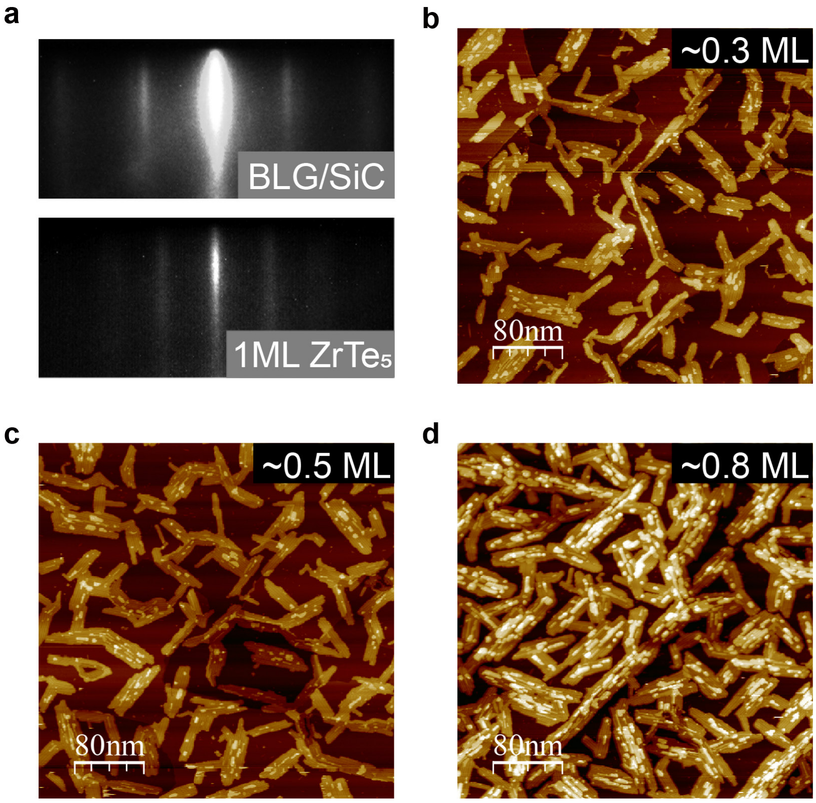

Supplementary Figure 1 Epitaxial $ZrTe_5$ monolayers grown on the BLG/SiC(0001) substrate. (a) RHEED patterns collected on the bare bilayer graphene (BLG)/SiC substrate (top panel) and epitaxial $ZrTe_5$ monolayer with the coverage of ~1.0 ML (bottom panel). (b,c,d) Large-scale STM images (400 × 400 nm^2) taken on the surface of $ZrTe_5$ monolayer on BLG/SiC substrate at various coverages. The coverages are ~0.3 ML for (b), ~0.5 ML for (c) and ~0.8 ML for (d), respectively. Bias voltage U = +1.0 V, tunneling current I_t = 20 pA.

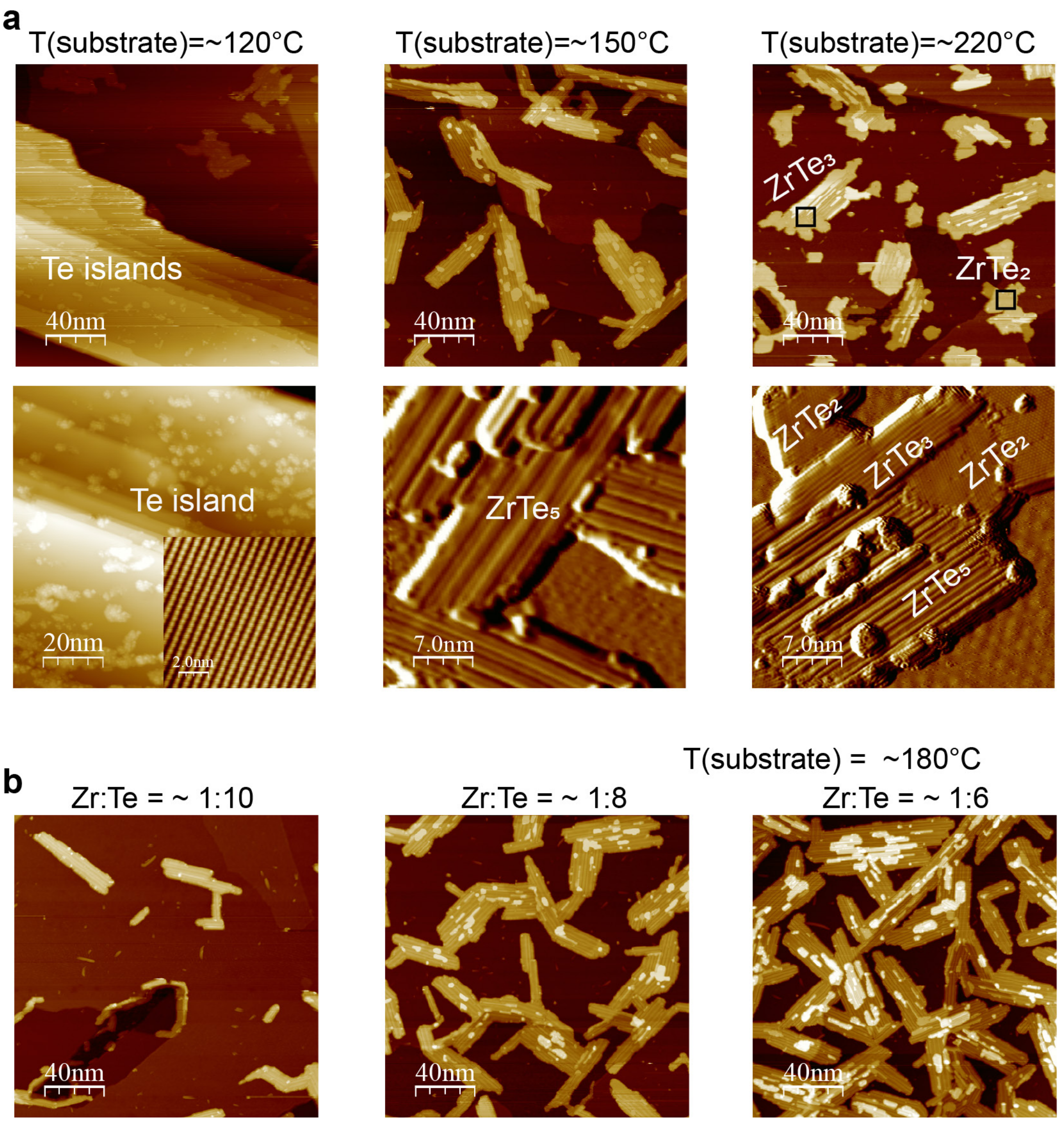

Supplementary Figure 2 Epitaxial $ZrTe_5$ monolayers grown at different substrate temperatures and flux ratios of Zr:Te. (a) Top panel: STM images (200 × 200 nm^2) of the $ZrTe_5$ on BLG/SiC grown at ~120°C, ~150°C, and 220°C respectively. Bias voltage U = +1.0 V, tunneling current I_t = 20 pA. Bottom panel: Zoom-in images of the top panel ones. (b) STM images of the $ZrTe_5$ on BLG/SiC grown at different Zr:Te flux ratios with a substrate temperature of ~180 °C. Bias voltage U = +1.0 V, tunneling current I_t = 20 pA.

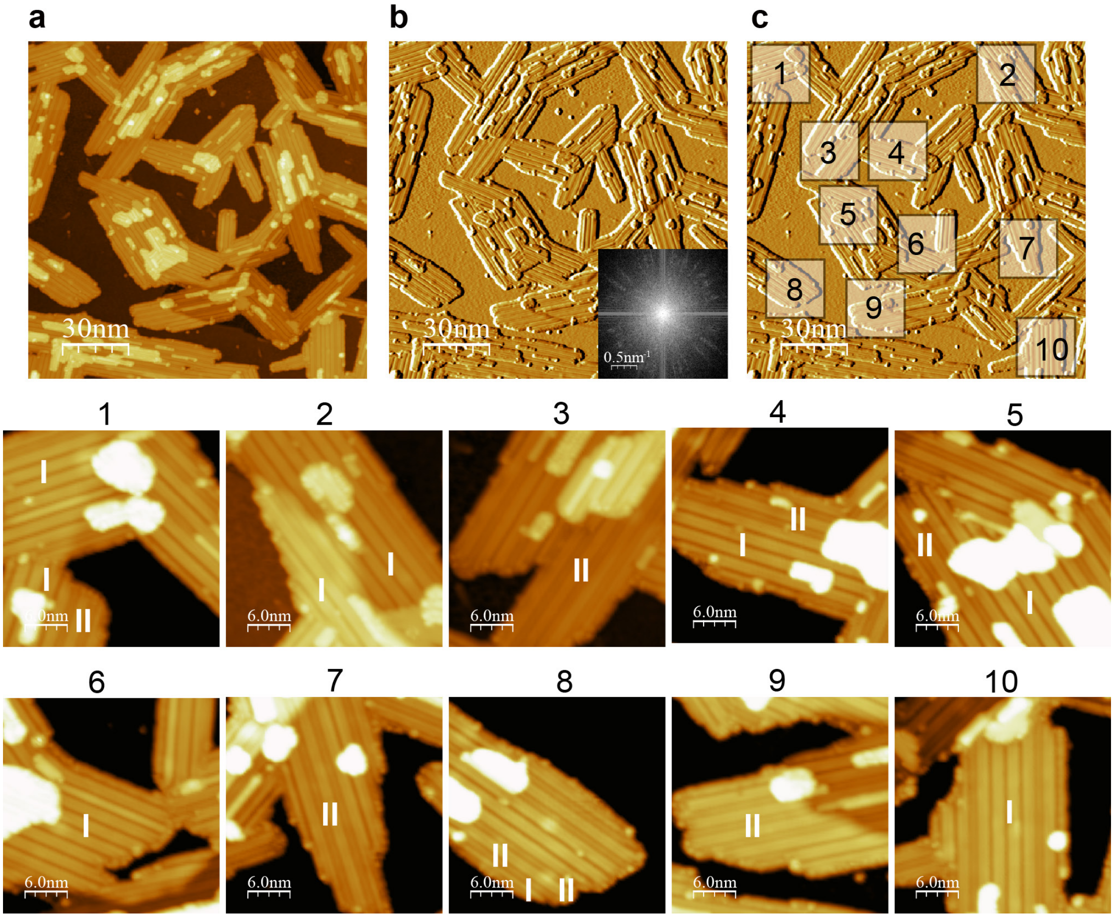

Supplementary Figure 3 (a) Large-scale STM image (150 × 150 nm^2) of the epitaxial $ZrTe_5$ monolayers on the BLG/SiC substrate. Bias voltage U = +1.0 V, tunneling current I_t = 20 pA. (b) Derivative image of (a). Inset: FFT image of (b) showing the orientation of the $ZrTe_5$ monolayers. (c) The same image of (b) with 10 labeled squares from "1" to "10". The zoom-in images (30 × 30 nm^2) of the labeled squares are depicted in the bottom with the same labels. The phases I and II regions are marked by "I" and "II" in the zoom-in images.

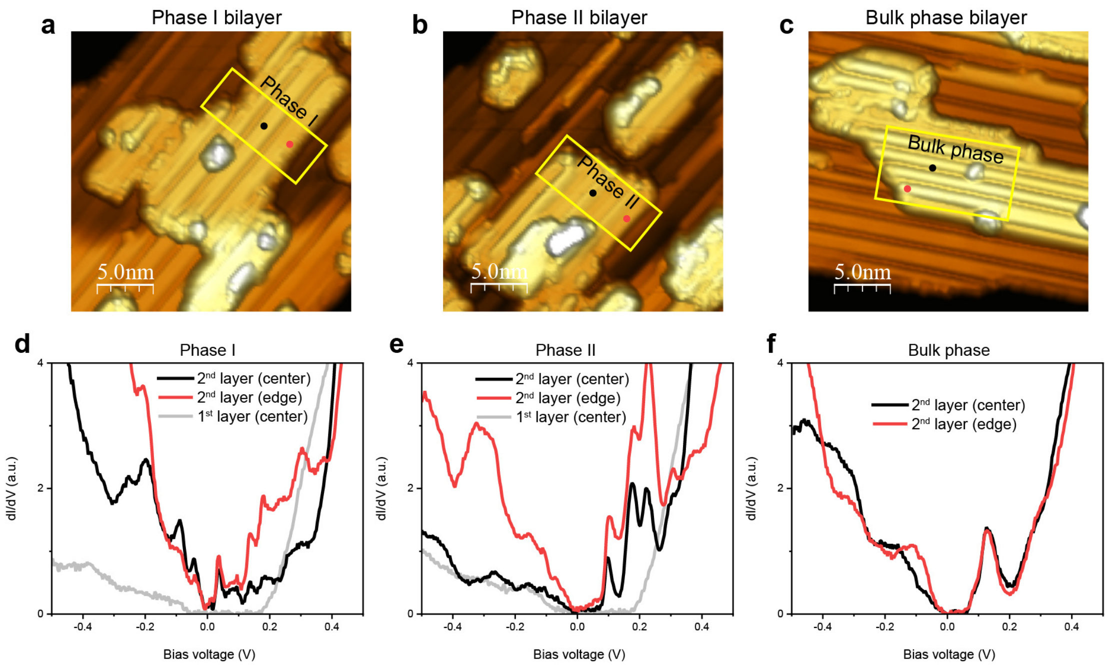

Supplementary Figure 4 (a)-(c) STM images (3d view: 25 × 25 nm^2) showing the bilayer $ZrTe_5$ islands with the second layer in phase I (a), phase II (b) and bulk phase (c), respectively. The yellow rectangles mark the second-layer $ZrTe_5$ regions, and underneath the second-layer regions are the first-layer $ZrTe_5$. The black and red dots mark the locations where the STS dI/dV spectra are taken. (d) STS dI/dV spectra taken at the center (black) and edge (red) of the second-layer $ZrTe_5$ in phase I. For comparison, the STS dI/dV spectrum taken at the first-layer $ZrTe_5$ of phase I is also plotted (gray). (e, f) STS dI/dV spectra taken on the second-layer $ZrTe_5$ in phase II and bulk phase, respectively.

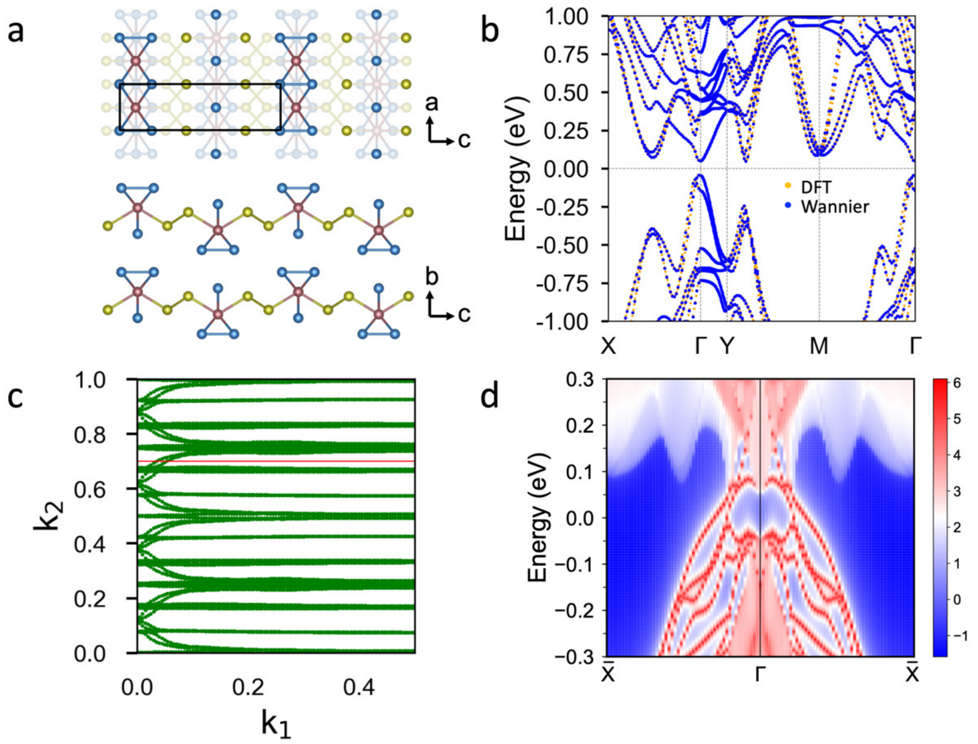

Supplementary Figure 5 (a) Top (upper panel) and side (lower panel) views of the crystal structure, (b) band structures obtained from the DFT calculations and Wannier interpolation, (c) Wilson loop, and (d) edge states of the $ZrTe_5$ homobilayer with AA stacking in bulk phase. The black rectangle in (a) represents the primitive unit cell.

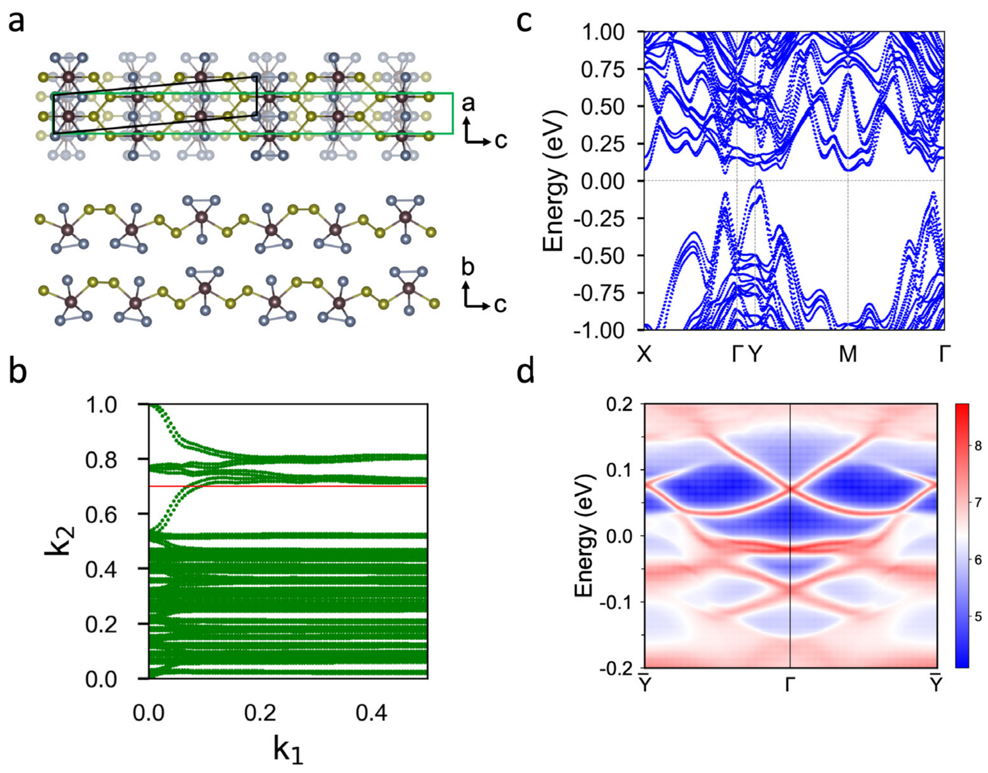

Supplementary Figure 6 (a) Top (upper panel) and side (lower panel) views of the crystal structure, (c) band structure obtained from the Wannier interpolation, (b) Wilson loop, and (d) edge states of the $ZrTe_5$ homobilayer with AA stacking in phase I. The black primitive unit cell in (a) is used for calculations.

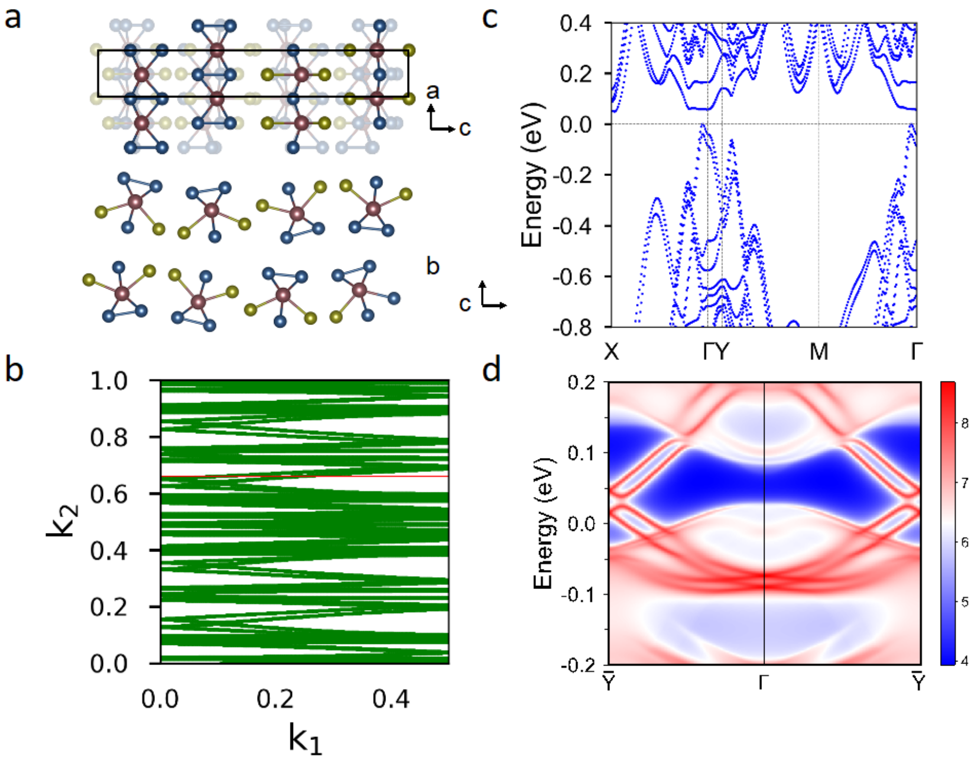

Supplementary Figure 7 (a) Top (upper panel) and side (lower panel) views of the crystal structure, (c) band structure obtained from the Wannier interpolation, (b) Wilson loop, and (d) edge states of the $ZrTe_5$ homobilayer with AB stacking in phase II. The black rectangle in (a) represents the primitive unit cell.

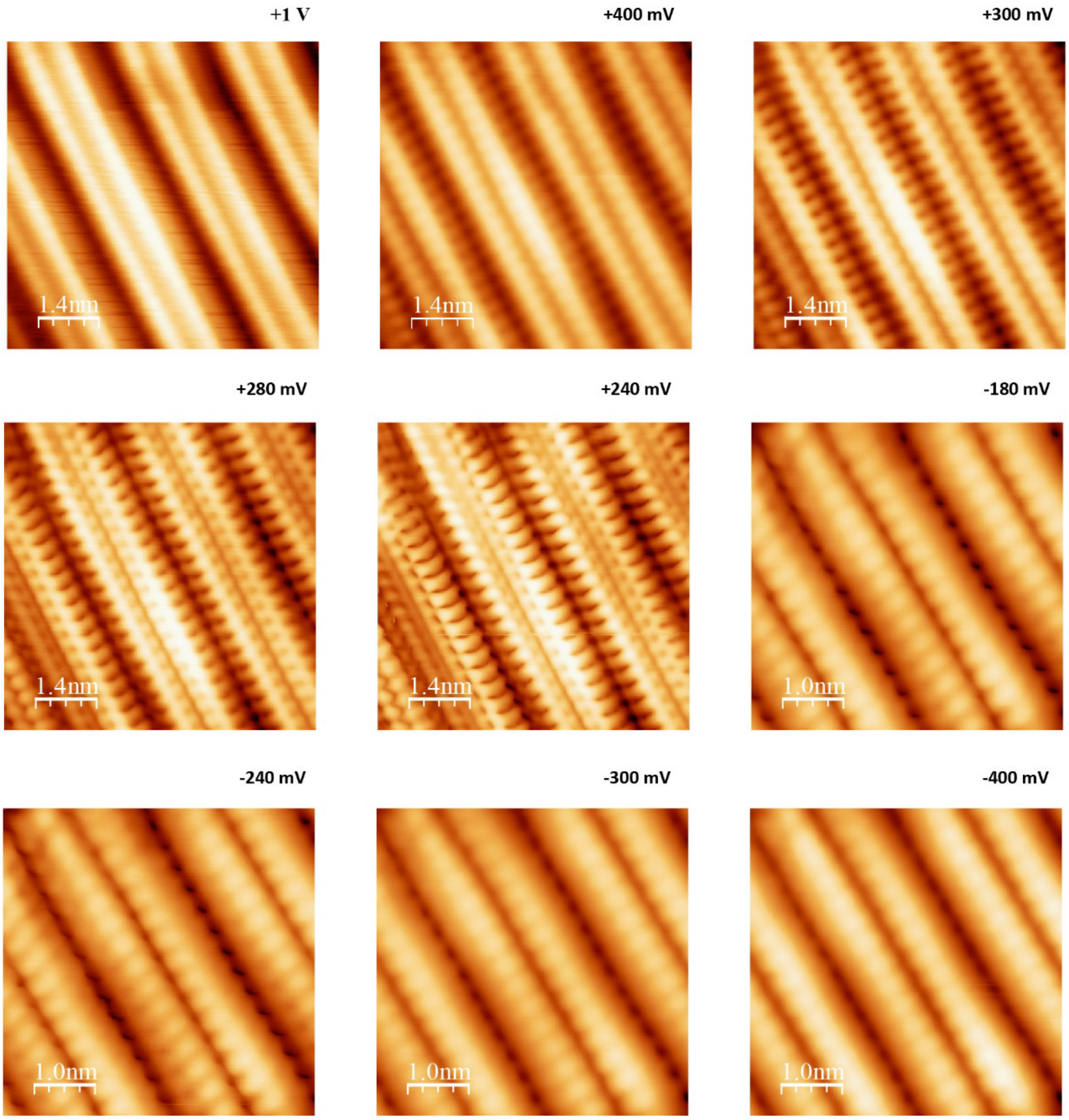

Supplementary Figure 8 Atomically resolved STM images (5 × 5 nm^2) of phase I taken at various voltages.

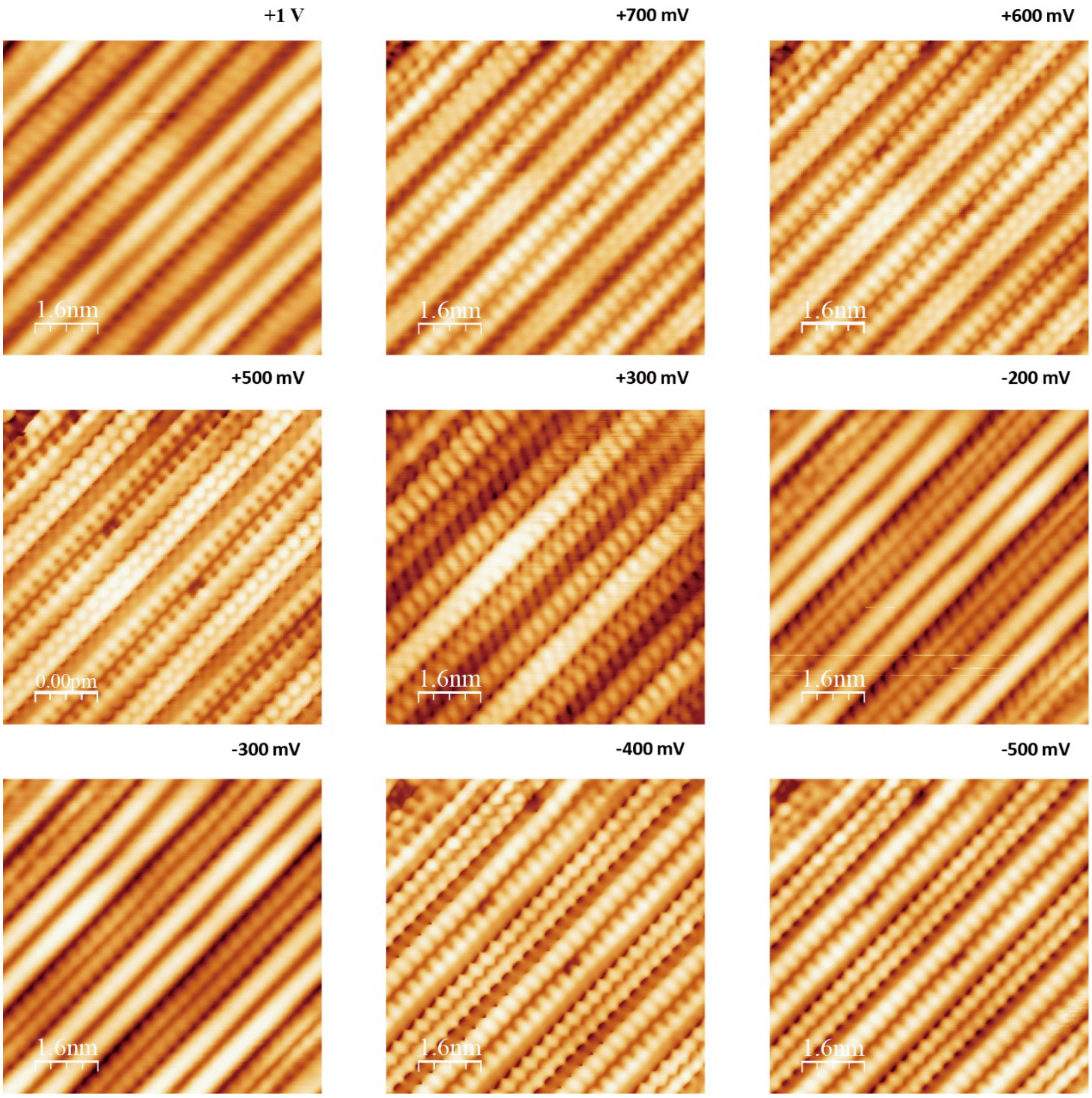

Supplementary Figure 9 Atomically resolved STM images (8×8 nm^2) of phase II taken at various voltages.

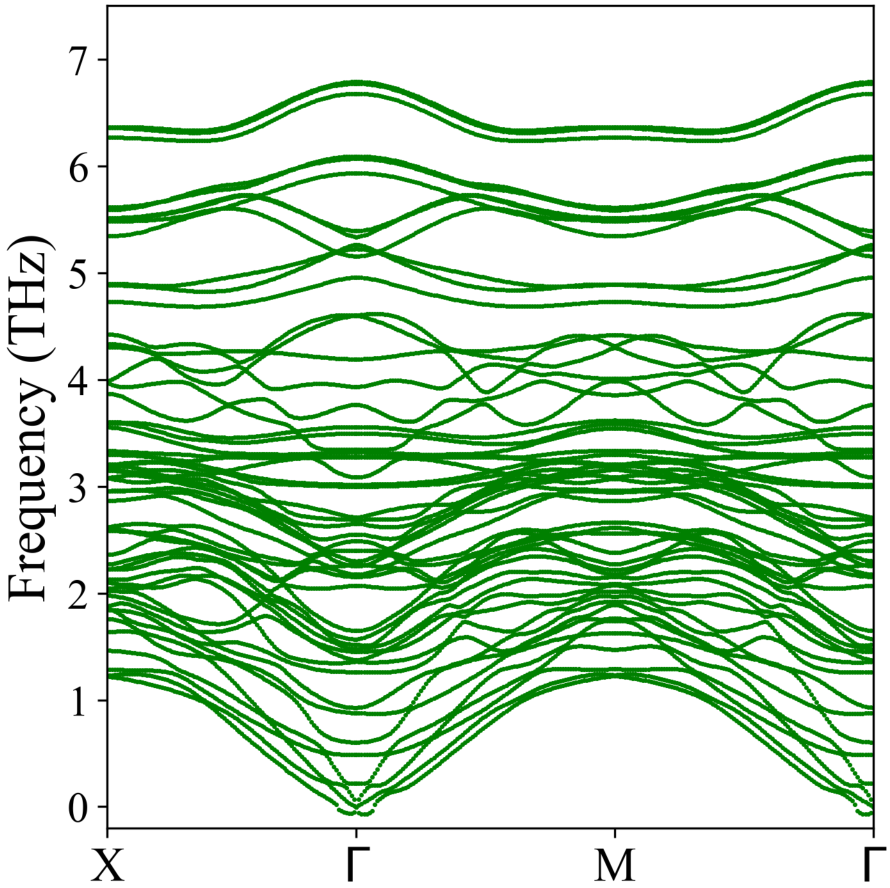

Supplementary Figure 10 Phonon spectrum of phase I with the ‘221’ configuration.

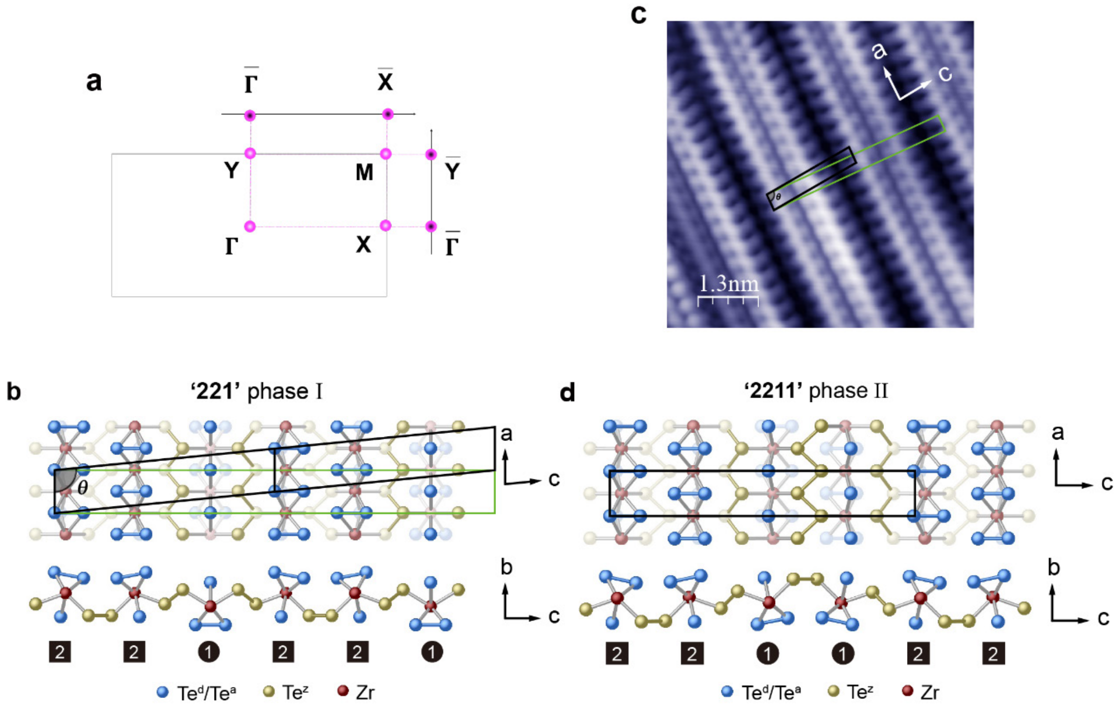

Supplementary Figure 11 Atomic structures of the epitaxial $ZrTe_5$ monolayer for phases I and II. (a) First Brillouin zone of phase I or II. (b) Top and side views of the crystal structure of phase I. The black parallelogram and green rectangle represent the primitive unit cell and orthorhombic cell, respectively. (c) Atomically resolved topographic images (6.5 × 6.5 nm^2) of a phase I region (U = +290 mV, I_t = 400 pA). The black parallelogram and green rectangle mark the primitive unit cell and extended orthorhombic cell of phase I, respectively. The angle of the parallelogram (θ) marked in (b) is ~95.57° and in (c) is ~95°. (d) Top and side views of the crystal structure of phase II. The rectangle represents the primitive unit cell.

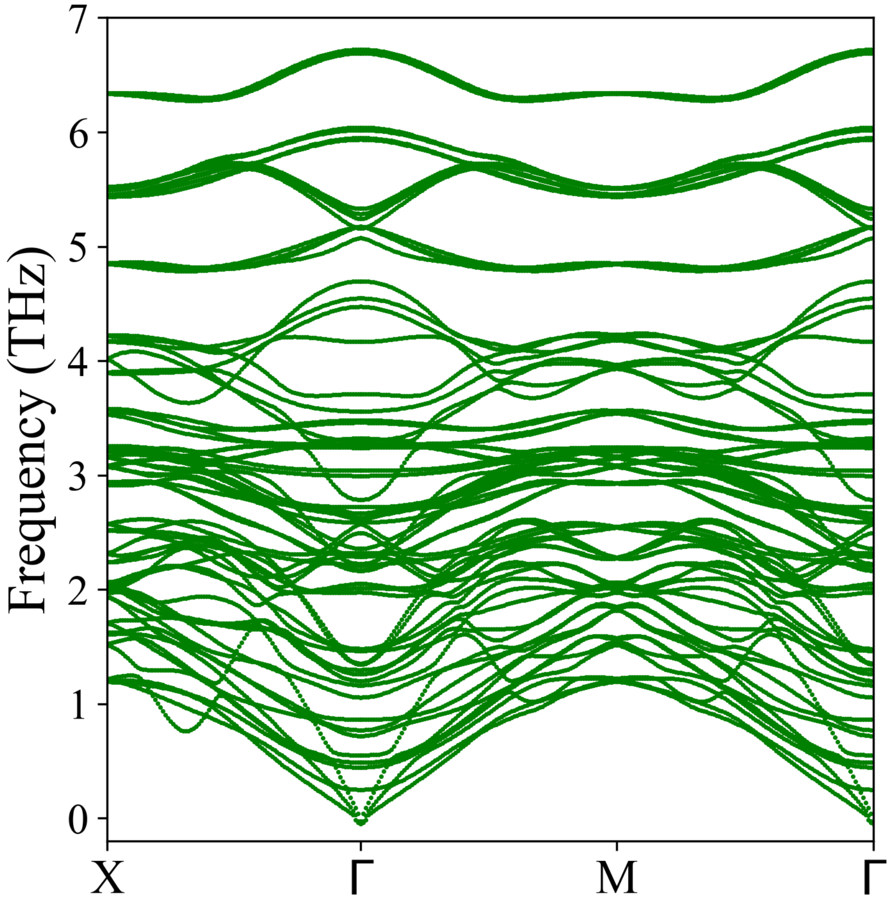

Supplementary Figure 12 Phonon spectrum of phase II with the ‘2211’ configuration.

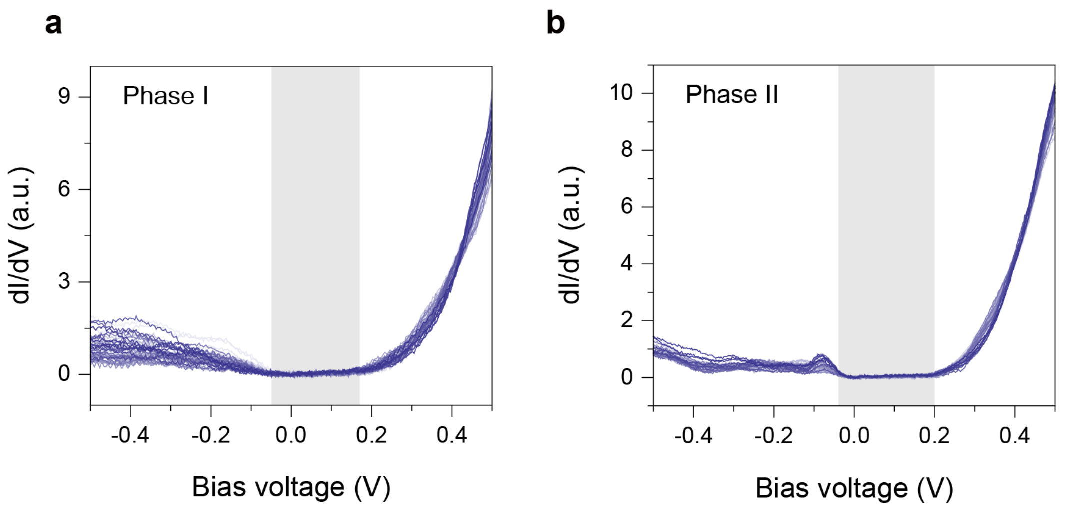

Supplementary Figure 13 Tunneling spectroscopic data taken on the epitaxial $ZrTe_5$ monolayers of phases I and II. (a) STS dI/dV spectra taken at different locations of the phase I terraces that are far away from the step edges. (U = 500 mV, I_t = 100 pA, U_{mod} = 8 meV). (b) STS dI/dV spectra taken at different locations of the phase II terraces that are far away from the step edges (U = 500 mV, I_t = 100 pA, U_{mod} = 8 meV). The gray columns marks the insulating gaps for both phases.

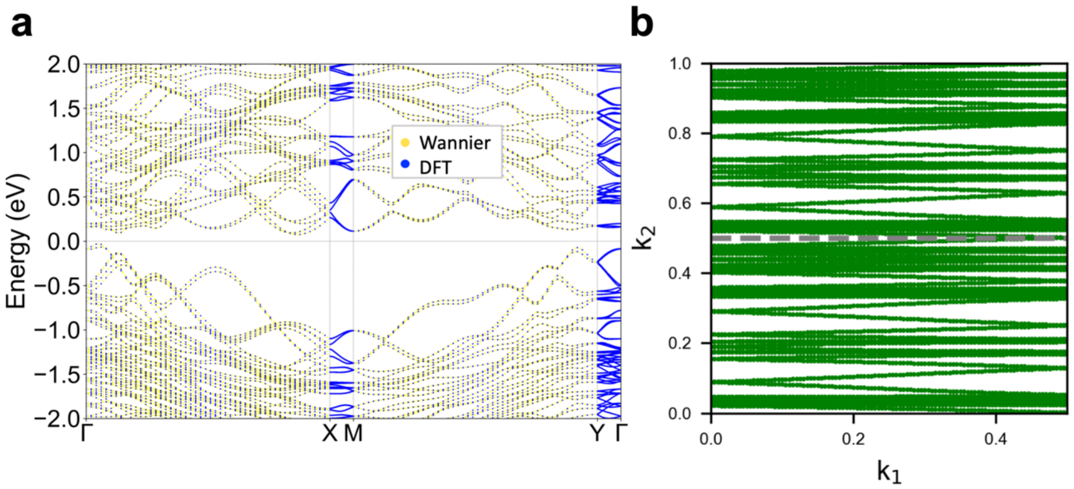

Supplementary Figure 14 (a) Band structures of phase I obtained from the DFT calculations and Wannier interpolation. (b) The corresponding Wilson loop.

To gain further insight into the topological properties of phase I (namely, the '221' configuration), we calculate the Z_2 topological invariant by tracing the evolutions of Wannier charge centers using a tight-binding Hamiltonian obtained with maximally localized Wannier functions. These calculations were performed using the Wannier90 and Wanniertools packages. Here, the Zr-*d* and Te-*p* orbitals were used for the initial projections to construct the tight-binding Hamiltonian. Based on this Hamiltonian, the obtained Wannier band structure shows a good agreement with the DFT one [see Supplementary Figure 14(a)], indicating that the selected orbitals are reasonable. Supplementary Figure 14(b) plots the Wilson loop of phase I, where the grey dashed line serves as a reference line. The Z_2 can be determined by counting the number of intersections between the evolution line and an arbitrary horizontal reference line. In this case, an odd number of crossings (the number is equal to 1) indicates that phase I is topologically nontrivial with $Z_2 = 1$.

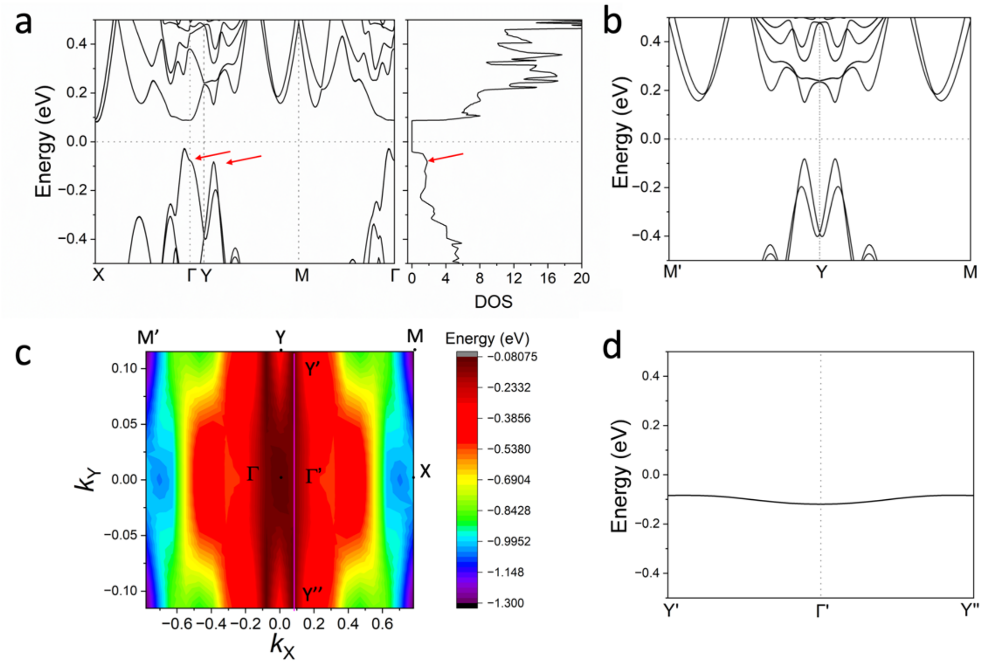

Supplementary Figure 15 (a) Band structure (left panel) and DOS (right panel) of phase II. (b) Band structure along the direction M′-Γ-M. (c) Energy dispersion relations of the highest valence band at the k_Z = 0 plane, where the energy eigenvalues are denoted by different colors. (d) Energy dispersion relation of the highest valence band along the pink line in (c).

We calculated the density of states (DOS) of phase II, as shown in the right panel of Supplementary Figure 15(a). It can be seen that a resonance also appears near -100 meV in the DOS (marked by the red arrow), which is consistent with the experimental results. To understand the physical reason of the presence of the resonance, we further examined the energy dispersions in the whole Brillouin zone. The energy dispersion relation of the highest valence band at the k_Z = 0 plane is plotted in Supplementary Figure 15(c). It is noted that there exit Rashba-type bands along the M′-Y-M direction [Supplementary Figure 15(b)] besides the bands at the Γ point. From Supplementary Figure 15(c) and (d), these Rashba bands are nearly flat along the Y′-Γ-Y″ direction, thereby resulting in a pronounced DOS near -100 meV. As a result, such a pronounced DOS in the electronic structure contributes to the experimentally observed resonance at -100 meV.

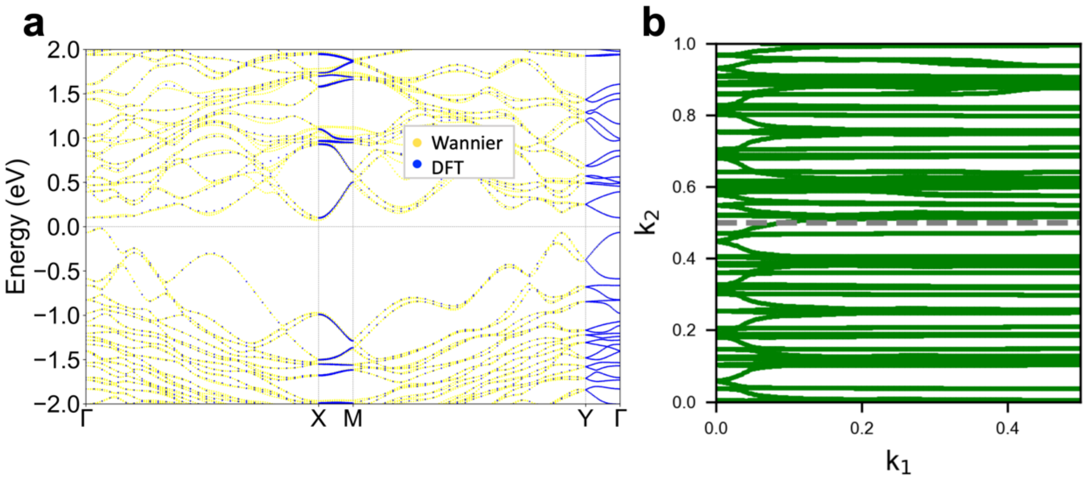

Supplementary Figure 16 (a) Band structures of phase II obtained from the DFT calculations and Wannier interpolation. (b) The corresponding Wilson loop.

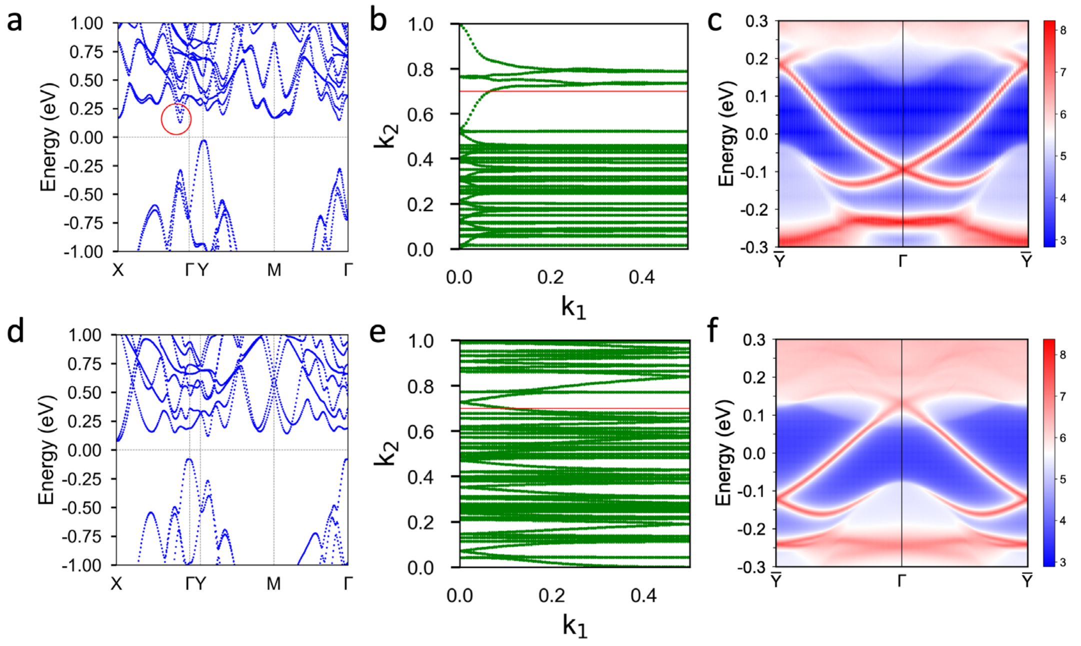

Supplementary Figure 17 (a) Band structure obtained from the Wannier interpolation, calculated with HSE06 hybrid functional, where the primitive unit cell in Fig. 2(c) is used, (b) the corresponding Wilson loop and (c) surface states for phase I. (d-f) Same with (a-c) but for phase II.

We have carried out additional first-principles calculations using the HSE06 hybrid functional with different mixing parameters (AEXX = 0.1, 0.2, 0.25, 0.3, and 0.5) to check the band gaps and topological properties of phase I and phase II. The largest band gaps are identified to be 160 and 157 meV for phase I and phase II, respectively, both with AEXX = 0.25 in the hybrid functional calculations. Supplementary Figure 17 plots the band structures, corresponding Wilson loop and edge states for both two phases. For phase II, the band gap increases by 47 meV, while for phase I, the band gap remains nearly unchanged, which may be because its conduction band minimum shifts to a lower location close to the Γ point (marked in the red circle in Supplementary Figure 17(a)). Overall, the calculated band gaps remain smaller than the experimental values. Within the HSE06 functional calculations, we also confirm that both phases persist as topologically nontrivial with $Z_2 = 1$ and exhibit topologically protected edge states [see Supplementary Figure 17(b,c,e,f)].

Supplementary Table 1. Summarized crystal parameters of bulk $ZrTe_5$ monolayered '21' phase, '221' phase I, and '2211' phase II, in experiment and theory.

Samples	*a* (Å)	*c* (Å)	*b* (Å)	∠*a*oc (º)	Gap (meV)	Group
$ZrTe_5$-exp. [1]	3.988	13.724	14. 530	90		C_{mcm} (63)
'21' phase-theo. [2]	4.036	13.843		90	100	P_{mmn} (59)
'221' phase (phase I)-exp.	3.9	21.0		95	220	
'221' phase (phase I)-theo.	4.017	20.691		95.57	169	C_{mm2} (35)
'2211' phase (phase II)-exp.	3.9	26.8		90	240	
'2211' phase (phase II)-theo.	4.020	27.235		90	110	P_{bmc} (57)

Supplementary Table 2. Summarized crystal parameters, band gaps, space group, with/without inversion symmetry, and Z_2 values for the three homobilayers, which are the respective most stable configurations in bulk phase, phase I, and phase II.

Samples	*a* (Å)	*c* (Å)	∠*a*oc (º)	Gap (meV)	Space group	Inversion symmetry	Z_2
Bulk bilayer	4.0	13.81	90	91	Pmma	yes	0
Phase-I bilayer	4.02	20.66	95.57	46	Cm	no	0
Phase-II bilayer	4.02	27.09	90	50	P2/m	yes	0

Supplementary References

1. Fjellvåg H, Kjekshus A. Structural properties of $ZrTe_5$ and $HfTe_5$ as seen by powder diffraction. *Solid State Communications* **60**, 91-93 (1986).

2. Weng H, Dai X, Fang Z. Transition-Metal Pentatelluride $ZrTe_5$ and $HfTe_5$: A Paradigm for Large-Gap Quantum Spin Hall Insulators. *Physical Review X* **4**, 011002 (2014).
